# Fibroblast Reprogramming in Gastrointestinal Cancer

**DOI:** 10.3389/fcell.2020.00630

**Published:** 2020-07-15

**Authors:** Maria-Theodora Melissari, Niki Chalkidi, Michalis E. Sarris, Vasiliki Koliaraki

**Affiliations:** Institute for Fundamental Biomedical Research, Biomedical Sciences Research Center “Alexander Fleming”, Vari, Greece

**Keywords:** cancer-associated fibroblasts, tumor microenvironment, synthetic activation, epigenetic reprogramming, metabolic reprogramming

## Abstract

Gastrointestinal cancers are a significant cause of cancer mortality worldwide and have been strongly linked with chronic inflammation. Current therapies focus on epithelial/cancer cells; however, the importance of the tumor microenvironment in the development and treatment of the disease is also now well established. Cancer-associated fibroblasts (CAFs) are a major component of the tumor microenvironment, and are actively participating in tumor initiation, promotion and metastasis. They structurally and functionally affect cancer cell proliferation, tumor immunity, angiogenesis, extracellular matrix remodeling and metastasis through a variety of signaling pathways. CAFs originate predominantly from resident mesenchymal cells, which are activated and reprogrammed in response to cues from cancer cells. In recent years, chronic inflammation of the gastrointestinal tract has also proven an important driver of mesenchymal cell activation and subsequent CAF development, which in turn are capable of regulating the transition from acute to chronic inflammation and cancer. In this review, we will provide a concise overview of the mechanisms that drive fibroblast reprogramming in cancer and the recent advances on the downstream signaling pathways that regulate the functional properties of the activated mesenchyme. This new mechanistic insight could pave the way for new therapeutic strategies and better prognosis for cancer patients.

## Introduction

CAFs are an essential component of the tumor microenvironment and accumulating evidence supports their substantial contribution to cancer development and progression (Kalluri, 2016; Kobayashi et al., 2019; Sahai et al., 2020). They originate predominantly from tissue-resident fibroblasts that are activated in response to signals from cancer cells and the tumor microenvironment. Additional cellular sources, such as bone marrow-derived mesenchymal stromal cells (BM-MSCs), fibrocytes, as well as epithelial and endothelial cells have also been reported. Fibroblast activation includes increased proliferation, changes in their physicochemical properties, such as shape alteration and increased contractility, and the production of a variety of effector molecules. These include cytokines and chemokines, extracellular matrix (ECM) components and remodeling enzymes, growth factors, metabolites and signaling molecules that mediate CAF functions to support cancer growth, metastasis and resistance to therapy (Kalluri, 2016; Kobayashi et al., 2019; Sahai et al., 2020). The increased insight into CAF functions and their association with poor prognosis in cancer patients has brought into focus the potential of CAF targeting for cancer treatment. It is thus interesting to consider the possibility of the reversal of CAF reprogramming as a promising therapeutic strategy in cancer, although potential anti-tumor CAF properties should also be taken into account (Gieniec et al., 2019).

**FIGURE 1:**
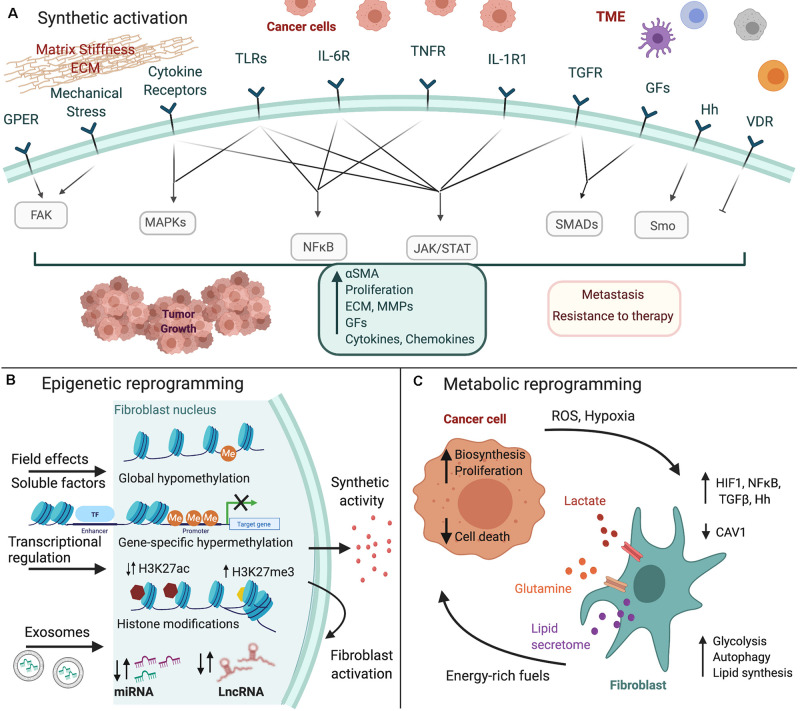
Fibroblast reprogramming in gastrointestinal cancer. Cancer cells and the tumor microenvironment (TME) induce the **(A)** synthetic activation of fibroblasts to CAFs, as well as their **(B)** epigenetic and **(C)** metabolic reprogramming. Major inducing signals and downstream pathways that regulate these processes are shown. The figure was created with BioRender.com.

In this review, we summarize current knowledge on how fibroblasts are converted to CAFs, particularly focusing on gastrointestinal cancers, including colorectal cancer (CRC), pancreatic ductal adenocarcinoma (PDAC), hepatocellular carcinoma (HCC), and gastric cancer (GC). We specifically analyze the three major types of CAF reprogramming, namely synthetic, epigenetic and metabolic, and focus on the signals and downstream molecular pathways that regulate this transition (Figure 1).

## Fibroblast Reprogramming: Types and Underlying Molecular Mechanisms

### Synthetic Activation

A variety of signals, such as growth factors, cytokines, chemokines, Toll-like receptor (TLR) and Hedgehog ligands, mechanical forces and ECM components activate fibroblasts to produce effector molecules that are responsible for CAF functions (Figure 1A; Kalluri, 2016; Kobayashi et al., 2019; Sahai et al., 2020). TGFβ and IL-1 are probably the most ubiquitous and well-characterized such stimuli.

TGFβ is a dominant effector in all gastrointestinal cancers and mediates the conversion of fibroblasts to CAFs. Its importance is highlighted by the association of a CAF-specific TGFβ signature with poor prognosis, immune cell exclusion and resistance to immunotherapy in CRC (Calon et al., 2015; Tauriello et al., 2018). The pleiotropic effects of TGFβ encompass changes in fibroblast adhesion through the production of collagens and fibronectin, changes in cell shape through αSMA overexpression, increased proliferation and increased synthetic activity, including the production of ECM enzymes, growth factors, chemokines, and cytokines (Calon et al., 2012; Hawinkels et al., 2014; Ishimoto et al., 2017; Biffi et al., 2019). TGFβ functions predominantly by downstream activation of SMADs and JAK/STAT signaling pathways, as shown in CRC and liver fibrosis (Calon et al., 2012; Tang et al., 2017). In contrast, it downregulates IL-1R1 and blocks the JAK/STAT cascade in PDAC fibroblasts, favoring thus the generation of CAFs with a myofibroblastic phenotype (Biffi et al., 2019). Notably, it also promotes epithelial-to-mesenchymal and endothelial-to-mesenchymal transition, as well as the recruitment of bone marrow-derived mesenchymal cells (BM-MSCs) and fibrocytes (Polyak and Weinberg, 2009; Quante et al., 2011). Other growth factors can also regulate CAF reprogramming either alone or in combination with TGFβ. For example, PDGF has been shown to drive recruitment and activation of fibroblasts in CRC and GC and its blockade leads to reduced tumor growth and metastasis (Ostman, 2004; Kitadai et al., 2006; Kodama et al., 2010; Sumida et al., 2011; Manzat Saplacan et al., 2017). It also complements the function of TGFβ on both pancreatic (PSC) and hepatic (HSC) stellate cells to promote their proliferation and migration (Dong et al., 2004; Cadamuro et al., 2013).

Inflammation is a significant predisposition factor for the initiation and progression of gastrointestinal cancers. The abundance of inflammatory signals mediates reciprocal interactions between cancer cells, stroma and immune cells to accelerate the development of an inflammatory TME and the phenotypic switch of CAFs (Greten and Grivennikov, 2019). Recently, Biffi and Tuveson (2020) highlighted the importance of IL-1 signaling in shaping CAF functions in PDAC, by showing that tumor-derived IL-1α antagonizes TGFβ signaling and stimulates the production of a cytokine cascade, including LIF, IL-6 and CXCL8 (Biffi et al., 2019). This acts in an autocrine manner to activate the JAK/STAT3 pathway in CAFs, resulting in a positive feedback loop that leads to high IL-1R1 expression and inflammatory CAF formation (Biffi et al., 2019). IL-1β also drives tumor fibrosis and cancer cell proliferation, survival and chemoresistance in PDAC through the IRAK4-NFκB pathway (Zhang et al., 2018). The IL-1-NFκB axis plays also an important role in the activation or survival of HSCs, at least in liver fibrosis (Gieling et al., 2009; Pradere et al., 2013). Both IL-1α and IL-1β increase CAF motility in GC through the regulation of Rhomboid 5 homolog 2 (RHBDF2), which promotes TGFβR1 cleavage by ADAM17 (Ishimoto et al., 2017). In the intestine, they induce cytokine and prostaglandin production by intestinal mesenchymal cells, which promote inflammation and can support the establishment of a cancer stem cell niche (Li et al., 2012; Scarpa et al., 2015). Interestingly, IL-1β was recently shown to drive the activation of subsets of PDGFRα^+^ fibroblasts, contributing thus to EGF-dependent serrated polyp formation in the mouse cecum (He et al., 2019). In contrast, in vivo deletion of IL-1R1 in *ColVI*^*Cre*+^ mesenchymal cells had no effect in either Apc-driven spontaneous or inflammation-induced intestinal carcinogenesis (Koliaraki et al., 2019). These studies highlight both the diverse and opposing roles of IL-1 agonists in cancer and their potential distinct functions in different fibroblast or CAF subpopulations (Voronov et al., 2013).

TNF and members of the IL-6 family are also important inflammatory inducers of fibroblast activation. TNF has been shown to act synergistically with IL-1α to promote proinflammatory gene expression in PSCs through NFκB activation (Biffi et al., 2019). Accordingly, in vivo inhibition of TNF was able to reduce desmoplasia in mice, which was associated with decreased PSC viability (Zhao et al., 2016). In addition, in vitro studies ascribe a pro-fibrogenic role for TNF on HSCs, as it promotes myofibroblast survival and activation through NFκB activation, while iRhom2-mediated inhibition of TNFR signaling protects against liver fibrosis (Tarrats et al., 2011; Pradere et al., 2013; Bonnardel et al., 2019; Sundaram et al., 2019). IL-6 and IL-11 were recently also shown to play a role in fibroblast activation in CRC through STAT3 activation and subsequent expansion of activated fibroblasts and the induction of a proangiogenic profile, which drove colorectal carcinogenesis in vivo (Heichler et al., 2019). Similarly, IL-6 was sufficient to induce the trans-differentiation of normal fibroblasts to CAFs via STAT3 phosphorylation and downstream activation of a Twist1/CXCL12 axis (Lee et al., 2015).

Besides these major cytokine signals, other cytokines and chemokines, such as IL-33 and CXCL12 also contribute to fibroblast activation. In humans and mouse models of intestinal cancer, cancer cell-derived IL-33 activates fibroblasts and promotes the expression of ECM components and growth factors associated with intestinal tumor progression (Maywald et al., 2015). Accordingly, IL-33 drives hepatic fibrosis through activation of HSCs via MAPK signaling (Tan et al., 2018). CXCL12 can promote epithelial-to-mesenchymal transition (EMT), recruit BM-MSCs in gastric cancer and drive the expansion of αSMA^+^ myofibroblasts and Gremlin 1-expressing mesenchymal stem cells (Quante et al., 2011; Shibata et al., 2013). Other immune cell-derived inflammatory cytokines, such as IL-17, IL-22, IL-31, IL-4 and IL-13, have also been shown to activate quiescent tissue-resident fibroblasts during inflammation or fibrosis, although their role in CAF reprogramming is not yet clear (Andoh et al., 2007; Tsuchida and Friedman, 2017).

Beyond cytokines and chemokines, innate immune signals have emerged as an additional stimulus for CAF reprogramming, linking further the inflammatory TME with fibroblast activation and cancer progression (Koliaraki et al., 2020). CAFs express innate immune receptors and respond to produce effector molecules that affect tumourigenesis in gastrointestinal tumors. This is mediated through downstream activation of MAPK and NFκB signaling pathways, which independently of the stimuli have been shown to play an important role in the synthetic reprogramming of CAFs in colorectal cancer (Koliaraki et al., 2012, 2015, 2019; Henriques et al., 2018). We recently showed that innate activation of intestinal mesenchymal cells through TLR4/MyD88 pathway in the *Apc^*min/*+^* mouse model resulted in the production of pro-tumourigenic and immunomodulatory effector molecules, while it also resembled the gene expression profile of human CRC CAFs (Koliaraki et al., 2019). Similarly, TLR9 activation has been shown to lead to PSC reprogramming and the production of CCL11 to support PDAC tumor growth (Zambirinis et al., 2015).

The Hedgehog (Hh)/Smoothened (Smo) signaling pathway has also been shown to regulate CAF activation in gastrointestinal cancers, although it seems to exert opposite functions depending on the organ affected. Several studies have shown that genetic deletion or pharmacological inhibition of Hh signaling leads to depletion of the PDAC-associated stroma and enhanced drug delivery, xenograft growth inhibition, as well as reduction of HSC activation and concomitant liver fibrosis (Yauch et al., 2008; Olive et al., 2009; Michelotti et al., 2013; Swiderska-Syn et al., 2014). In contrast, Hh activity was found reduced in human CRC and stroma-specific Hh activation markedly decreased tumor load and progression in a mouse model of CRC partly via the modulation of BMP signaling (Gerling et al., 2016). Notably, clinical trials using Hh inhibitors in patients with CRC or PDAC have so far failed, indicating that stromal heterogeneity, compensatory mechanisms and therapy resistance could interfere with CAF reprogramming approaches in solid tumors (Berlin et al., 2013; Kim et al., 2014; Catenacci et al., 2015).

Besides driving CAF activation, other pathways have been shown to suppress or reverse it and could be promising candidates for therapeutic interventions. A prominent example is the vitamin D receptor (VDR), which acts as a modulator of CAF reprogramming in gastrointestinal cancers (Ferrer-Mayorga et al., 2017). Mechanistically, it has been shown to suppress PSC activation, resulting in stromal remodeling and increased intratumoral gemcitabine effects in PDAC, while interaction between VDR and p62/SQSTM1 suppressed HSC activation and liver cancer (Sherman et al., 2014; Duran et al., 2016). Another such example is the activation of p53 by Nutlin-3a, which can reprogram activated PSCs to quiescence (Saison-Ridinger et al., 2017).

Finally, beyond soluble mediators, the biophysical properties of the TME have been also implicated in CAF activation and the induction of a synthetic phenotype. Mechanical stress induces collagen overexpression and crosslinking, fiber rearrangement, ECM deposition and degradation by fibroblasts, which can thus mediate the remodeling of the ECM and increase matrix stiffness (Mohammadi and Sahai, 2018). Matrix stiffness and the resulting mechanical stress further activates fibroblasts in a continuous self-promoting loop resulting in cancer cell proliferation and migration. Several studies in gastrointestinal inflammation and cancer propose that these stimuli activate fibroblasts through FAK, MRTF-SRF, and YAP-TEAD signaling pathways, leading to increased αSMA expression and the regulation of cytoskeletal dynamics (Johnson et al., 2013, 2014; Foster et al., 2017). Many of these effects are also dependent on TGFβ, Rho and ROCK signaling (Zhao et al., 2007; Johnson et al., 2014). Accordingly, HSCs sense mechanical stress through integrins, GPCRs and DDRs, activating Rho, YAP, PAK1, and JAK2/PI3K/AKT-MYOCD, respectively (Martin et al., 2016; Kang, 2020). Interestingly, another mechanosensor, the G protein-coupled estrogen receptor (GPER) shows tumor-restricting capacity, as it acts through Rho/myosin axis and YAP deactivation to inhibit the ability of PSCs and HSCs to remodel the ECM (Cortes et al., 2019a, b).

### Epigenetic Reprogramming

Epigenetic abnormalities, including changes in DNA methylation, abnormal patterns of histone modifications and post-transcriptional regulation through micro and long non-coding RNAs support genetic changes in cancer cells to drive tumor initiation and progression. Similar genetic mutations driving CAF differentiation are rare (Qiu et al., 2008; Bianchi-Frias et al., 2016). However, CAFs maintain their properties in vitro, indicating that they could also be epigenetically modified to a stably activated cell state (Figure 1B; Kalluri, 2016).

Both global and gene-specific changes in DNA methylation patterns have been detected in stromal cells and shown to affect tumor growth and progression. GC CAFs display global hypomethylation of DNA along with hypermethylation at a subset of genes, such as *HOXB6* (Jiang et al., 2008). Global alterations in 5-methylcytosine and 5-hydroxylmethylcytosine play an important role in HSC activation and concomitant liver fibrosis (Page et al., 2016). PDAC CAF differentiation is accompanied by decreased cytosine methylation and increased hydroxymethylation in response to cancer cell-derived lactate and subsequent CAF metabolic alterations that drive activation of the demethylase TET (Bhagat et al., 2019). An example of gene-specific methylation change includes the cancer cell-induced methylation and concomitant downregulation of the SOCS1 gene in PDAC CAFs, which enhanced cancer cell growth through the STAT3/IGF1 axis (Xiao et al., 2016). Increased PGE2 production in response to *H. pylori* infection was shown to drive hypermethylation of miR-149, increased IL-6 secretion and CAF activation in GC (Li et al., 2015). Interestingly, CRC stromal cells showed hypermethylation of the *SEPT9* gene, which was temporally subsequent to epithelial cells, suggesting that DNA methylation in CAFs could be attributed to field effects from cancer cells (Wu et al., 2007). These examples highlight the role of cancer cells in the epigenetic reprogramming of fibroblasts, although more research is necessary to define the molecular mechanisms involved in this process.

Evidence on the regulation of histone modifications in stromal cells and their role in fibroblast activation during carcinogenesis is still limited. Nevertheless, genome-wide H3K27me3 analyses of primary GC CAFs revealed loss of H3K27me3 in genes involved in the maintenance of the stem cell niche, including WNT5A, the inhibition of which was shown to suppress cancer cell growth and migration (Maeda et al., 2020). Similarly, the histone acetyltransferase P300 was shown to mediate stiffness-induced activation of HSCs by altering the acetylation status of H3K27Ac, at least on the CXCL12 gene promoter (Dou et al., 2018). Additional genome-wide screenings in combination with functional in vivo studies on gastrointestinal cancer are necessary to delineate the role of the “histone-code” on the activation of stromal cells.

Changes at the levels of multiple miRNAs have also been implicated in the reprogramming of fibroblasts in gastrointestinal cancers. For example, activated PSCs differentially express 84 miRNAs (Masamune et al., 2014), while culture-induced HSC activation resulted in the deregulation of 259 miRNAs (Coll et al., 2015). Among deregulated mi-RNAs, miR-21 is of particular interest, as it is involved in the activation of fibroblasts in colorectal and pancreatic cancer, as well as oesophageal squamous cell carcinoma through mechanisms that include activation of the TGFβ pathway and the promotion of the metabolic reprogramming of CAFs (Bullock et al., 2013; Li et al., 2013; Nouraee et al., 2013; Chen et al., 2018). These changes can be either the result of complex deregulated transcriptional and post-transcriptional networks in differentiating fibroblasts or the consequence of miRNA transfer from cancer cells mainly through exosomes. Concerning the latter, several studies have indeed confirmed this mechanism also for gastrointestinal cancers. For example, pancreatic cancer cells can reprogram normal fibroblasts to CAFs through secreted microvesicles containing miR-155, miR-1246, and miR-1290 (Pang et al., 2015; Masamune et al., 2018). In addition, metastatic hepatocellular carcinoma cells secrete exosomal miR-1247-3p that targets B4GALT3, which in turn activates β1 integrin-mediated NFκB signaling in fibroblasts (Fang et al., 2018). Finally, long noncoding RNAs have been described as regulators of stromal activation in HSCs (Zhou et al., 2016). These can act through a circuit comprising also miRNAs, such as GAS5 that restrains hepatic fibrosis by targeting miR-23a through the PTEN/PI3K/Akt signaling pathway (Dong et al., 2019) and HOTTIP which promotes the activation of HSCs via the downregulation of miR-148a (Li et al., 2018).

### Metabolic Reprogramming

Alterations in cancer cell energetics are now considered a hallmark of cancer (Hanahan and Weinberg, 2011; Faubert et al., 2020). The most prominent such change is a shift of glucose metabolism towards aerobic glycolysis versus mitochondrial oxidative phosphorylation (OXPHOS), a phenomenon that is known as the “Warburg effect”. This allows cancer cells to take advantage of glycolytic intermediates and activate the biosynthesis of macromolecules and organelles that support rapid growth and proliferation (Vander Heiden et al., 2009; Faubert et al., 2020). Fibroblasts in the surrounding tumor microenvironment also exhibit a similarly altered metabolism towards aerobic glycolysis, which leads to the release of energy-rich fuels, mainly lactate, but also pyruvate, glutamine and ketone bodies (Wu et al., 2017). These are transferred from CAFs to cancer cells through MCT4 and MCT1 transporters, respectively, where they are used to replenish the TCA cycle, to support OXPHOS and biosynthesis pathways maximizing proliferation and reducing cell death. This phenomenon is termed the “Reverse Warburg effect” (Wilde et al., 2017). In line with this, MCT1 and MCT4 levels were found upregulated in CRC and were associated with low survival in patients with CRC and gastrointestinal stromal tumors (GISTs) (Lehuede et al., 2016; Martins et al., 2016). Similarly, PDAC CAFs displayed diverse expression of the hypoxic marker carbonic anhydrase X and MCT4 and altered metabolic properties, which supported the invasiveness of cancer cells and were correlated with shorter patient survival (Knudsen et al., 2016).

Besides glucose metabolism, CAFs also display a lipid metabolic shift. For example, activated PSCs showed a remodeled and increased lipid secretome and produced lysophospatidylcholines, which support membrane lipid synthesis, while they were further converted to LPA via autotaxin enzymatic activity to facilitate tumor growth (Auciello et al., 2019). Similarly, CAFs in CRC accumulated fatty acids and phospholipids via an increase in fatty acid synthase (FASN), and were then transferred to cancer cells to induce their migration (Gong et al., 2020).

CAFs are also characterized by increased autophagy, which generates recycled nutrients from broken down organelles that in turn are used by cancer cells to cover their needs. For example, in PDAC, cancer cell-induced autophagy in CAFs leads to the secretion of non-essential amino acids, and specifically alanine, which in turn fuels the TCA cycle and lipid biosynthesis in the cancer cells (Sousa et al., 2016). In CRC, co-culture of fibroblasts and cancer cells resulted in an upregulation of oxidative stress-related enzymes and autophagy genes and the downregulation of CAV1 in fibroblasts that in turn promoted cancer cell proliferation (Zhou et al., 2017).

Mechanistically, CAF metabolic reprogramming and autophagy are induced mainly by reactive oxygen species (ROS) and hypoxia, which through downstream activation of HIF1 and NFκB promote the metabolism of glucose to lactate and glutamate and mediate the loss of caveolin-1 (CAV1) (Figure 1C) (Martinez-Outschoorn et al., 2010). Inflammatory mediators can also induce autophagy in fibroblasts through NFκB signaling, providing evidence for immune regulation of metabolic reprogramming, similarly to their synthetic activation (Martinez-Outschoorn et al., 2011). TGFβ also mediates CAFs’ metabolic reprogramming either through the downregulation of CAV1 or the upregulation of autophagy/mitophagy inducers. It acts by downregulating isocitrate dehydrogenase 1 (IDH1), leading thus to an increase of glutamine metabolism and α-ketoglutarate (α-KG) concentration, which in turn suppresses CAV1 expression (Hou et al., 2017). CAV1 is a crucial regulator of CAFs’ metabolic switch and its inhibition is sufficient to activate fibroblasts, impair mitochondrial function and induce a glycolytic switch in fibroblasts through the upregulation of glycolytic enzymes (Sotgia et al., 2012). Finally, the Hh pathway has been shown to play a significant role in the reprogramming of quiescent HSC to myofibroblasts during liver fibrosis, and potentially CAF differentiation, through the activation of aerobic glycolysis and lactate accumulation (Chen et al., 2012). Additionally, Hh signaling together with YAP were found to induce glutaminolysis, concomitant activation of HSC and fibrosis progression (Du et al., 2018).

### Reprogramming and Heterogeneity of CAFs

Differences in reported CAF functions, including tumor-promoting and restraining roles have long led to the hypothesis that distinct CAF subpopulations could exist within tumors (Kalluri, 2016; Gieniec et al., 2019; Biffi and Tuveson, 2020). Representative such examples include the tumor-promoting effects of CAF depletion or suppression in PDAC mouse models (Rhim et al., 2014; Özdemir et al., 2014). However, low cell abundancy and lack of analytical techniques have until recently hindered the functional characterization of these potentially different subtypes. Advances in single-cell analysis technologies have increased our understanding of tumor heterogeneity, including that of the microenvironment and specifically CAFs. Concerning gastrointestinal cancers, a recent single-cell transcriptomic analysis of mouse and human PDAC identified three CAF subsets, namely inflammatory (iCAFs), myofibroblastic (myCAFs) and antigen-presenting CAFs (apCAFs) (Elyada et al., 2019). Mechanistic studies using organoids and mouse models showed that iCAFs express inflammatory markers and are located within the desmoplastic stroma, while myCAFs are αSMA positive and adjacent to tumor cells. Importantly, they are activated by different stimuli, IL-1α and TGFβ, respectively, the spatial distribution of which can regulate the swift from one CAF state to the other (Ohlund et al., 2017; Biffi et al., 2019). apCAFs express MHC class II and CD74 and can induce T-cell receptor (TCR) ligation in CD4^+^ T cells in an antigen-dependent manner, while they can be also converted to myofibroblasts (Elyada et al., 2019). These studies offer compelling evidence to support the idea that CAF reprogramming depends on the availability of stimuli in the surrounding microenvironment and may thus define CAF phenotypic and functional heterogeneity, although the CAFs’ diverse cellular sources could also contribute, as shown for other types of cancer (Raz et al., 2018).

Single-cell RNA transcriptomic analyses have also been performed for colorectal and head and neck cancer. These revealed the presence of normal fibroblasts along with two CAF subsets, a myofibroblastic αSMA^+^ and an ECM-expressing FAP^+^ population, although further functional characterisation of these is still missing (Li et al., 2017; Puram et al., 2017). Nevertheless, these studies indicate common diversity and potentially similar subpopulations in gastrointestinal tumors.

## Discussion

In this review, we have provided a concise overview of the molecular mechanisms underlying fibroblast reprogramming in gastrointestinal cancers. This is especially important as CAF phenotype reversal has been proposed as a potential therapeutic strategy in cancer (Valkenburg et al., 2018; Chen and Song, 2019). Both soluble factors and mechanical cues drive the reprogramming of fibroblasts through the activation of downstream signaling pathways in fibroblasts, indicating the impact of fibroblast localization in the growing tumor. Multiple inducers and mechanisms underlying the synthetic activation of CAFs have already been identified, but similar research on the epigenetic and metabolic reprogramming of CAFs is still limited. Additional mechanistic insights into these processes could help identify novel targets for therapeutic approaches, as well as diagnosis and patient stratification. Notably, new targets should be in the future assessed under the prism of the emerging concepts of CAF heterogeneity that is defined by potential different cell sources, location, and available stimuli.

## Author Contributions

M-TM, NC, and MS wrote sections of the manuscript and prepared the figure. VK critically revised the manuscript. All authors read and approved the submitted version.

## Conflict of Interest

The authors declare that the research was conducted in the absence of any commercial or financial relationships that could be construed as a potential conflict of interest.
